# Effect of the 2020 hypersensitivity pneumonitis guideline on the pathologic diagnosis of interstitial pneumonia

**DOI:** 10.1038/s41598-023-35986-9

**Published:** 2023-06-08

**Authors:** Mutsumi Ozasa, Andrey Bychkov, Yoshiaki Zaizen, Kazuhiro Tabata, Wataru Uegami, Yasuhiko Yamano, Kensuke Kataoka, Takeshi Johkoh, Hiroshi Mukae, Yasuhiro Kondoh, Junya Fukuoka

**Affiliations:** 1 Department of Pathology Informatics, Nagasaki University Graduate School of Biomedical Sciences, 1-12-4 Sakamoto, Nagasaki, 852-8523 Japan; 2 Department of Pathology, Kameda Medical Center, Kamogawa, Chiba Japan; 3 Department of Respiratory Medicine and Allergy, Tosei General Hospital, Seto, Aichi Japan; 4 Department of Radiology, Kansai Rosai Hospital, Amagasaki, Hyogo Japan; 5 Department of Respiratory Medicine, Nagasaki University Graduate School of Biomedical Sciences, Nagasaki, Japan

**Keywords:** Diseases, Respiratory tract diseases

## Abstract

It was reported that the 2020 guideline for hypersensitivity pneumonitis (HP) might result in the overdiagnosis of fibrotic HP (fHP). fHP and other types of interstitial pneumonias have several overlapping characteristics, and a high diagnostic concordance rate of fHP is rarely obtained. Therefore**,** we investigated the impact of the 2020 HP guideline on the pathological diagnosis of cases previously diagnosed as interstitial pneumonia. We identified 289 fibrotic interstitial pneumonia cases from 2014 to 2019 and classified them into four categories according to the 2020 HP guideline: typical, probable, and indeterminate for fHP and alternative diagnosis. The original pathological diagnosis of 217 cases were compared to their classification as either typical, probable, or indeterminate for fHP according to the 2020 guideline. The clinical data, including serum data and pulmonary function tests, were compared among the groups. Diagnoses changed from non-fHP to fHP for 54 (25%) of the 217 cases, of which, 8 were typical fHP and 46 were probable fHP. The ratio of typical and probable fHP cases to the total number of VATS cases was significantly lower when using transbronchial lung cryobiopsy (p < 0.001). The clinical data of these cases bore a more remarkable resemblance to those diagnosed as indeterminate for fHP than to those diagnosed as typical or probable. The pathological criteria in the new HP guidelines increase the diagnosis of fHP. However, it is unclear whether this increase leads to overdiagnosis, and requires further investigation. Transbronchial lung cryobiopsy may not be helpful when using the new criteria to impart findings for fHP diagnosis.

## Introduction

The classification of fibrotic interstitial pneumonia (IP) is vital for predicting a prognosis and deciding a treatment plan^1^. However, the diagnosis of fibrotic IP remains challenging even for experts specializing in respiratory diseases. Studies have reported high interobserver variability in the identification of fibrotic hypersensitivity pneumonitis (fHP)^2,3^. One study, based on their diagnostic algorithm, suggested that nearly half of the current hypersensitivity pneumonitis (HP) cases have potentially been misdiagnosed as idiopathic IP^4^. Their results illustrate the diagnostic variables between teams. There has been no consensus on the criteria for the pathological diagnosis of fHP, and most pathologists diagnose fHP based on textbook definitions and literature reviews^5–7^.

The treatment and diagnosis of fHP is difficult. HP is always included in the differential diagnosis of fibrotic IP, although the standardization of the diagnosis of fHP has been long anticipated^8,9^. Thus, the formulation of specific guidelines providing diagnostic criteria for differentiating between HP and other IPs was highly desirable. Recently, leading authorities in this field developed diagnostic criteria in the Modified Delphi Survey^10^, and two guidelines for HP were published in 2020^11,12^.

According to this guideline, the pathological diagnoses of HP are divided into three categories: “typical HP,” “probable HP,” and “indeterminate for HP.” The guideline also indicates alternative diagnosis not as a separate category but as a descriptive condition in the same table. The final multidisciplinary discussion diagnosis (MDD) using the suggested algorithm would lead to the identification of HP when pathological criteria point toward typical or probable HP and the clinical and radiological features do not clearly indicate HP. In such cases, the recommendation will be to treat the patient for HP. This process illustrates the critical value of correct pathological diagnosis.

If the idiopathic pulmonary fibrosis (IPF) and HP guidelines are used simultaneously, the decision to select the more suitable of the two guidelines would be difficult in some cases^3^. In that sense, the distinction between usual interstitial pneumonia (UIP)/IPF and fHP, a fairly common scenario, becomes challenging. In addition, fHP cannot be easily distinguished histopathologically from diseases that also exhibit patterns of UIP, such as connective tissue diseases^3,14^.

A recent study reported the possibility that about half of the cases diagnosed as IPF by MDD, according to the fHP guideline, may be overdiagnosed as fHP^15^. That study also showed that a pathological diagnosis significantly impacts the overall diagnosis. At present, no study has investigated the influence of the histological criteria of the 2020 HP guideline^11^ on the diagnosis of fibrotic IP. In the present study, focusing on the pathological domain, we applied the HP guideline to fibrotic IP cases, and compared the pathological diagnoses based on the new guideline to the original pathological diagnoses.

## Methods

This study was conducted in accordance with the tenets of the Declaration of Helsinki and approved by the Nagasaki University Hospital Clinical Research Ethics Committee (No. 20101918). Informed consent was obtained in the form of opt-out methodology. Cases of video-assisted thoracoscopic surgery (VATS) biopsy and transbronchial lung cryobiopsy (TBLC) in the pathology consultation database of interstitial lung diseases were used, and a keyword search for the terms “fibrotic interstitial pneumonia” or “interstitial fibrosis” was performed for the cases sent from a single pulmonary center. All pathological diagnoses based on the IPF guidelines and findings from each case were reviewed and confirmed by two pulmonary pathologists on our team specializing in interstitial lung disease. The evaluated pathological findings included airway-centered fibrosis (ACF) with or without peribronchiolar metaplasia (PBM), poorly formed granulomas including interstitial giant cells with/without cholesterol clefts, lymphoid follicles with germinal centers, plasmacytosis, aspirated particles, and necrotizing granulomas, based on the new HP guideline^11^. Cases with either of the latter four findings were considered those of alternative diseases and excluded from further analysis. ACF is based on the 2020 HP guideline, and PBM may or may not be present in fibrotic lesions. In VATS and TBLC, multiple and one or more ACFs were considered ACF positive, respectively. Although granulomas are mainly found in the peribronchiolar interstitium, as shown in the reference cited in the guideline, we judged a case as granuloma positive if multiple granulomas were present, even if only in the pleura or in the air space^16^.

Patients were classified into four categories according to the pathological criteria of the 2020 HP guideline: typical fHP, probable fHP, indeterminate for fHP, and alternative diagnosis. The categorization based on the 2020 HP guideline was compared with the original pathological diagnosis^11^.

Our original criteria for the diagnosis of HP were based on renowned studies in the field^5–7^. As such, we diagnosed HP when there were multiple pathological findings listed below and no findings indicative of other diseases (e.g., collagen disease or aspiration): non-necrotizing poorly formed granulomas in the interstitium, interstitial giant cells with/without cholesterol clefts, peribronchiolar interstitial inflammation, airway-centered accentuation and diffuse cellular infiltration. We also applied pathologic criteria from the IPF guidelines and corresponded HP diagnosis in cases fitting the criteria for possible UIP or not UIP, based on the 2011 IPF guidelines,or indeterminate for UIP or alternative diagnosis, based on the 2018 IPF guidelines^17,18^.

Clinical information was extracted from all cases. A comparative study was conducted between the group in which the diagnosis of HP did not change, and the group in which the original diagnosis changed upon application of the HP guideline. The clinical information collected at the moment of diagnosis included age, sex, smoking history, IgG, Krebs von den Lungen-6 (KL-6), bird antibody, and bronchoalveolar lavage fluid (BALF).

The patients were categorized into four groups. First, cases with an original diagnosis of HP, which was further judged to be typical or probable fHP according to the new guideline, were included in the HP + /HP + group; second, cases whose original diagnosis was not HP and changed to either typical or probable fHP according to the HP guideline, were included in the HP-/HP + group; third, cases which were not originally diagnosed as HP, which were judged to be indeterminate for fHP by the new guideline, were designated as the HP-/indeterminate group. Finally, cases diagnosed as HP, considered indeterminate for fHP based on the new guideline evaluation, were designated to the HP + /indeterminate group. The effects of the HP guideline on each modality, i.e., VATS, biopsy, and TBLC, were compared.

Statistical analyses were conducted using a Fisher's exact test, followed by a Chi-squared test, a one-way analysis of variance, and a multivariate Tukey analysis. An alluvial diagram was created to visualize the change in diagnosis using ggalluvial package in R (R Foundation for Statistical Computing, Vienna, Austria). Statistical analyses were performed using the open access EZR software^19^.

## Results

The total cases of VATS biopsy and cryobiopsy that we received from the participating institute between 2014 and 2019 were 371. Among the 371 cases, 289 cases were selected through keyword search using the terms “fibrotic interstitial pneumonia” or “interstitial fibrosis.”

### Disease distribution based on the 2020 HP guideline

**Figure 1:**
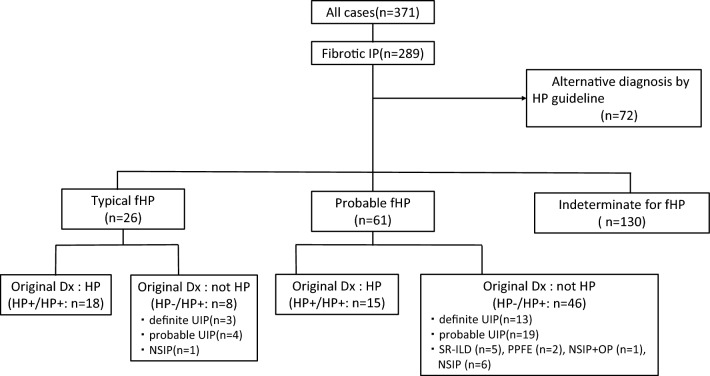
Flow diagram of the selection and application processes of the hypersensitivity pneumonitis guideline for cases with fibrotic interstitial pneumonia diagnosed using surgical lung biopsy and transbronchial lung cryobiopsy. fHP: fibrotic hypersensitivity pneumonitis; UIP: usual intersitital pneumonia, NSIP: nonspecific interstitial pneumonia, SR-ILD: smoking-related interstitial lung disease, PPFE: pleuroparenchymal fibroelastosis , OP: organizing pneumonia Dx: Diagnosis.

Seventy-two cases were histologically classified as alternate diseases by the guideline and were excluded due to strong histological autoimmune features, such as plasma cell infiltration or extensive lymphoid follicles, or due to the presence of sarcoid-like well-formed granulomas. These pathological diagnoses of the 72 cases included 70 IPs with autoimmune features and two sarcoid-like reactions. Eventually, 217 cases were classified into the following groups: typical fHP (*n* = 26), probable fHP (*n* = 61), and indeterminate for fHP (*n* = 130) using the 2020 HP guideline^11^ (Fig. 1).

Of the 26 typical fHP cases, 18 (69%) were originally diagnosed with HP. Eight cases (31%) had an original diagnosis other than HP, of which three, four, and one cases were originally diagnosed as having definite UIP, probable UIP, and nonspecific interstitial pneumonia (NSIP), respectively.

In the 61 probable fHP group, 15 cases (25%) were originally diagnosed with HP. While 46 cases (75%) had an original diagnosis other than HP, of which the principal diagnosis (*n* = 32) was definite or probable UIP, followed by NSIP (*n* = 6). Moreover, smoking-related interstitial lung disease, pleuroparenchymal fibroelastosis, and NSIP with organizing pneumonia were identified in five, two, and one cases, respectively (Fig. 1).

### Change of diagnosis based on the 2020 HP guideline

**Figure 2:**
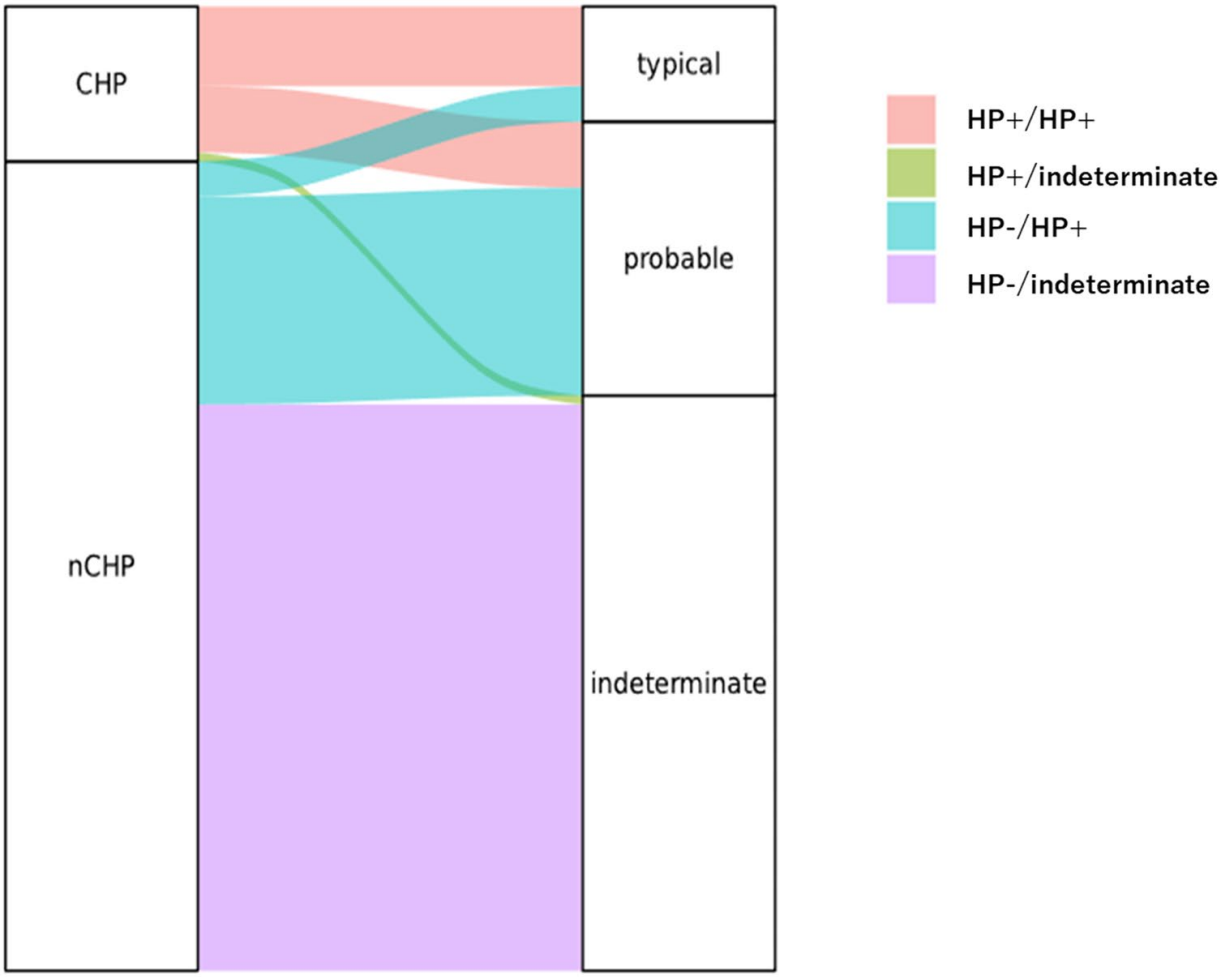
Alluvial plot to highlight the effect of the hypersensitivity pneumonitis guideline. The left and right columns show the original diagnosis and the diagnosis based on the 2020 HP guideline, respectively. CHP: chronic hypersensitivity pneumonitis; nCHP: not chronic hypersensitivity pneumonitis.

**Figure 3:**
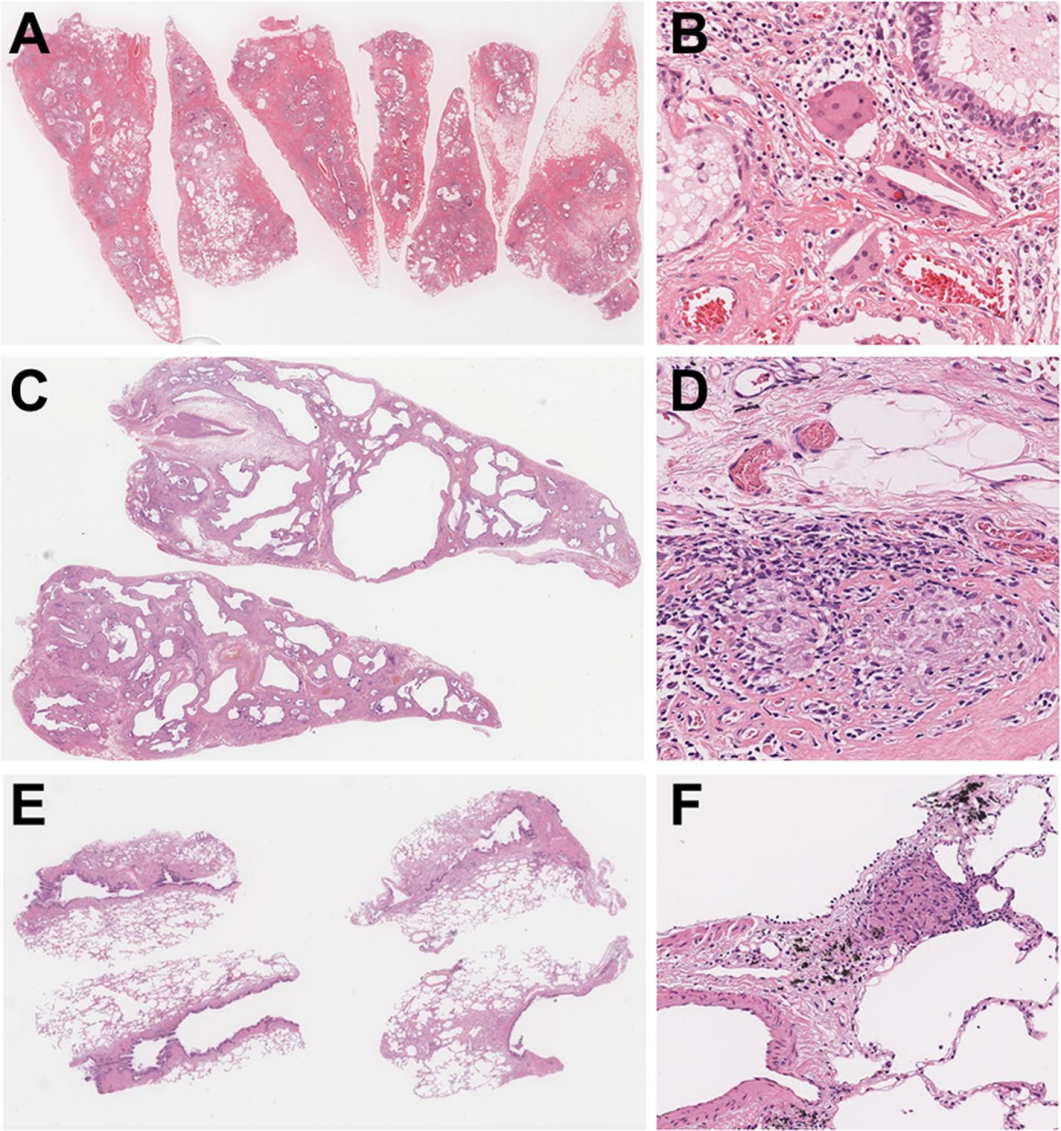
Cases recategorized as indeterminate for fibrotic hypersensitivity pneumonitis due to the presence of poorly formed granuloma but no airway-centered fibrosis. (**A**): Case of VATS biopsy shows diffuse end-stage lung (H&E, × 0.5). (**B**): Higher magnification of the same case presenting an accumulation of giant cells with aggregation of histiocytes and cholesterol clefts (H&E, × 20). (**C**): Scanning view of VATS biopsy showing end-stage honeycomb lung (H&E, × 0.5). (**D**): Higher magnification of the case C presenting poorly formed granulomas (H&E, × 20). (**E**): Scanning view of the cryobiopsy case showing interstitial fibrosis. Fibrosis is found around terminal airway which represents the central area inside the lung lobule (H&E, × 2). (**F**): Higher magnification of case E showing a focus of non-necrotizing granuloma (H&E, × 40). H&E: hematoxylin and eosin.

There were 33 cases in the HP + /HP + group. As shown in Fig. 1, the breakdown of the 33 cases showed 18 typical fHP and 15 probable fHP cases. The HP-/HP + group included 54 cases, the breakdown of which included eight typical fHP and 46 probable fHP cases. The remaining 130 cases belonged to the indeterminate group. There were three cases in the HP + /indeterminate group (Fig. 2). These three cases, including two VATS and one TBLC had non-necrotizing granulomas on the biopsy but lacked ACF and were categorized as indeterminate for fHP (Fig. 3A–F).

**Figure 4:**
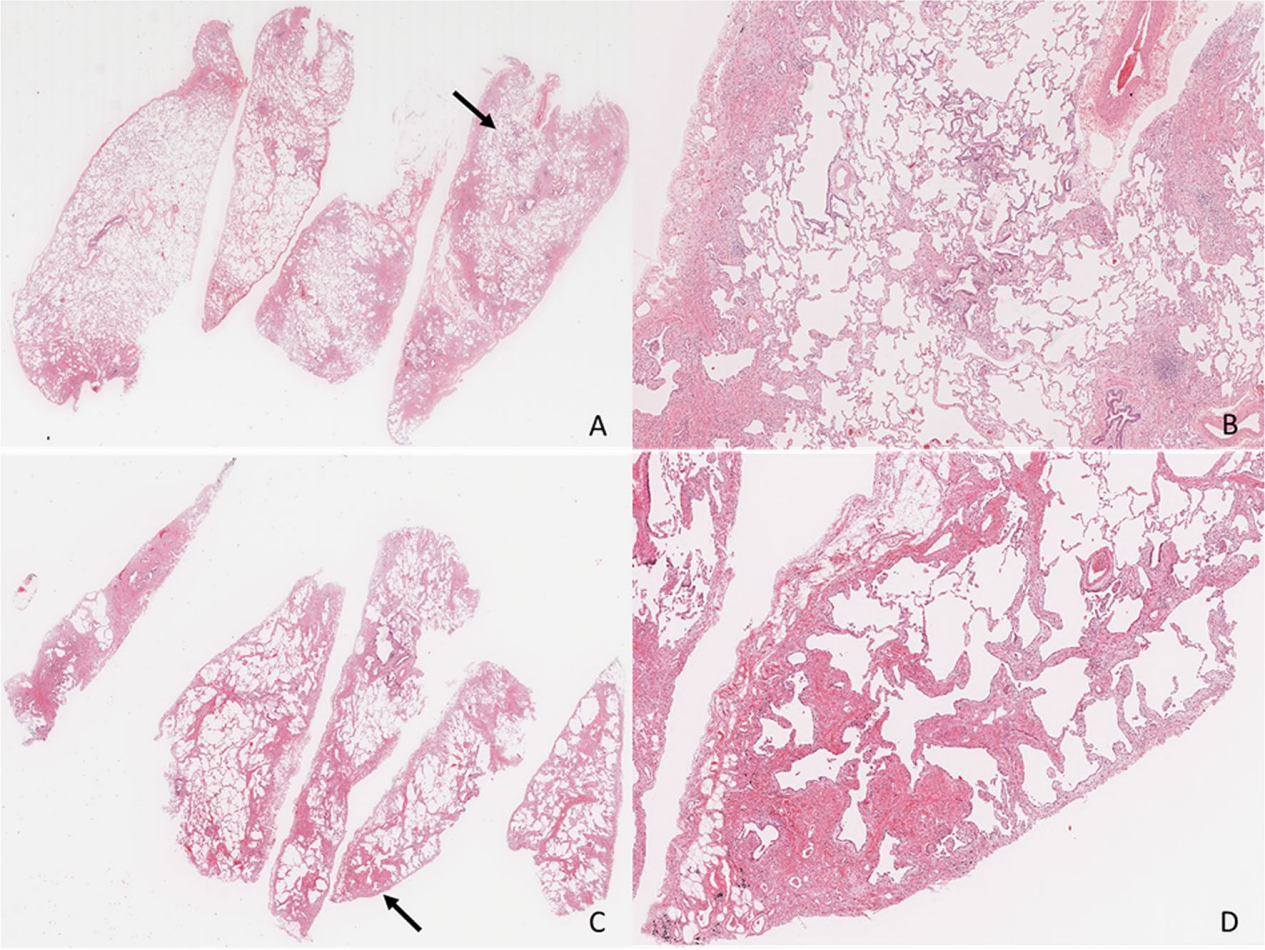
Two cases of fibrotic interstitial pneumonia where the diagnosis changed to fibrotic hypersensitivity pneumonitis after using the guideline. (**A** and **B**): Case 1, low and middle power views H&E, 0.5 × and 2 ×). (**C** and **D**): Case 2, low and middle power views (H&E, 0.5 × and 4 ×). Both cases show patchy fibrosis accentuated to the peripheral area inside the lobule. Note the presence of airway-centered fibrosis with peribronchiolar metaplasia (arrows). H&E: hematoxylin and eosin.

In the HP-/HP + group, eight cases were converted to typical fHP. The original diagnosis of the eight cases were definite UIP (n = 3), probable UIP (n = 4), and NSIP (n = 1) (Supplemental Fig. 1, 2, 3). Two of the eight cases had granulomas only in the pleura, and the changes in the lung fields were determined to be definite UIP in one case and definite fibrotic NSIP in the other (Supplemental Fig. 1A–E, 2A–E). We strictly used the criterion to evaluate granulomas suggesting HP for the ones located inside the interstitium; therefore, these granulomas were considered incidental findings were owing to their location. However, the 2020 HP guideline states that the location is not limited to the interstitium based on the observation by Castonguay et al.^11,16^ One case showed definite UIP without conspicuous inflammatory cell infiltration. The other five cases did not have granulomas with epithelioid cells. However, they all had collections of giant cells with a cholesterol cleft in the air space or interstitium (Supplemental Fig. 3A–C). The new guideline defines multinucleated giant cell aggregates as poorly formed granuloma. The location is not limited to the interstitium but can be in the air space or pleura; therefore, they were determined to be typical fHP. Of the 46 cases classified as probable fHP based on the guideline, most of the original diagnoses were definite UIP (n = 13) and probable UIP (n = 19), followed by five cases of smoking-related interstitial fibrosis diagnosed pathologically. These cases were reclassified as probable fHP due to the presence of ACF. On the other hand, ACF was frequently seen with emphysema and pigmented airspace macrophages, suggesting smoking-related changes in 25 HP-/HP + cases (Fig. 4A–D).

### Correlation to clinical data

Table 1 shows the comparison of clinical information of the three groups. Factors such as bird antigen (*p* = 0.018), lymphocyte fraction of BALF (*p* < 0.001), and CD4/CD8 (*p* < 0.001) showed statistically significant differences.

**Table 1:** Clinical information of the three groups.

Parameter	Original diagnosis / 2020 HP guideline diagnosis			*P-*value
	HP + / HP + (*n* = 33)	HP- / HP + (*n* = 54)	HP- / indeterminate (*n* = 127)	
Sex				
Female	10	12	44	0.26
Male	23	42	83	
**Age**	61.2 ± 12.3	62.7 ± 9.17	64.3 ± 9.02	0.34
**BI**	448 ± 483	595 ± 513	493 ± 616	0.43
Blood parameters				
IgG (mg/dL)	1503 ± 434	1594 ± 480	1493 ± 364	0.31
KL-6 (U/mL)	1735 ± 1452	1218 ± 889	1655 ± 1343	0.07
Bird antibody				
Positive	12	6	20	< 0.01
Negative	21	48	107	
BAL cells				
TC (× 10^5^)	2.37 ± 1.47	1.83 ± 1.28	14.4 ± 12.6	0.68
Mφ (%)	63.7 ± 27.3	82.7 ± 16.9	84.4 ± 80.8	0.25
Ly (%)	27.2 ± 26.1	11.8 ± 14.7	13.1 ± 16.2	< 0.01
CD4/8	4.52 ± 5.56	3.07 ± 2.07	2.42 ± 1.89	< 0.01

Dx: diagnosis, HP: hypersensitivity pneumonitis, BI: Brinkman index, KL-6: Krebs von den Lungen-6, BAL: bronchoalveolar lavage, TC: total cells, Mφ: macrophages, Ly: lymphocytes.

**Figure 5:**
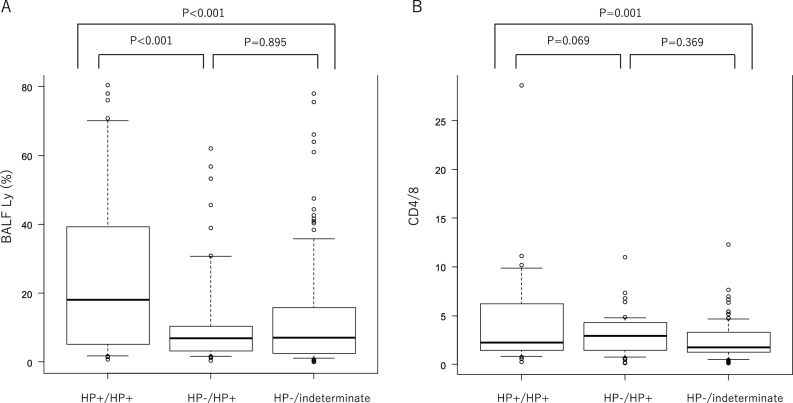
Comparison of the results of BALF among three groups based on the hypersensitivity pneumonitis guideline. (**A**): Comparison of the % of lymphocytes in the bronchoalveolar lavage fluid (BALF) in three groups. (**B**): Comparison of the CD4/8 in the BALF among three groups. CD4/CD8 in the BALF differed between the HP + /HP + and HP-/indeterminate groups (*P* < .001). Ly: lymphocytes HP: hypersensitivity pneumonitis.

The exposure to bird antigens differed significantly between the HP + /HP + and HP-/HP + groups and the HP + /HP + and HP-/indeterminate groups. The group with the highest exposure was HP + /HP + (36%), and the group with the lowest exposure was HP-/HP + (11%). The lymphocyte fraction in the BALF differed significantly between the HP + /HP + and HP-/indeterminate groups, and the HP + /HP + and HP-/HP + groups, but no significant difference was observed between the HP-/HP + and HP-/indeterminate groups (Fig. 5-A). CD4/CD8 in the BALF differed between the HP + /HP + and HP-/indeterminate groups (Fig. 5-B). Table 1 indicate mean with SD, Fig. 5A&B show median with IQR range.

### Analysis by sampling modality

Based on the HP guideline, 152 cases tested using VATS were classified into three categories of fHP; 23 were typical fHP, 56 were probable fHP, and 73 were indeterminate for fHP. Twenty-seven cases belonged to the HP + /HP + group, 52 to the HP-/HP + group, and 71 to the HP-/indeterminate group. TBLC was performed in 65 cases, of which three were diagnosed with typical fHP, five with probable fHP, and 57 were indeterminate for fHP. The quality of the 65 specimens was high in confidence in 61 cases and low in confidence in only four cases, indicating that 94% of the specimens were appropriate for evaluation. The ratios of typical fHP and probable fHP to the total number of VATS cases was 15% and 37%, respectively, and significantly lower at 4.6% and 7.7% for TBLC cases, respectively (*p* < 0.001).

## Discussion

The 2020 American Thoracic Society (ATS), the Japanese Respiratory Society (JRS), and the Asociación Latinoamericana de Tórax (ALAT) HP clinical practice guidelines paved the way for the standardization of diagnosing HP on an international scale, which, among other benefits, is expected to prevent a misdiagnosis with other IP entities ^11^. Reporting institutional experiences on the impact of the new guideline is essential to highlight the strengths and potential pitfalls of the new guideline. An accumulation of such reports will inevitably prompt further modifications and ensure the suitable development and evolution of the guideline. Recently, it was reported that the 2020 HP guideline might induce an overdiagnosis of HP cases by MDD ^11,15^. The prior study found that pathological diagnosis holds considerable sway over the diagnosis reached through MDD ^15^. MDD is, in a sense, a method to find a compromise between different diagnostic modalities. In this process, it is possible that an erroneous conjecture from one modality may impact the final diagnosis and push it in the wrong direction. To improve the diagnosis, we must seek ways to eliminate errors within each modality.

In this study, 87 (40%) out of 217 cases had typical fHP or probable fHP according to the HP guideline; while fHP was not confirmed in the original diagnosis; it was altered in 54 cases (25%), according to the 2020 HP guideline ^11^. These results suggest that the diagnosis in nearly one-fourth of all cases may be changed under the new HP guideline. Among the 87 cases, a total of 39 cases were originally diagnosed as definite UIP or probable UIP according to the IPF guideline. Currently, it is unclear what therapy those 39 patients whose diagnoses changed from IPF to fHP in the new guideline may benefit from. This requires further clinical investigation.

We compared cases belonging to the HP + /HP + and HP-/HP + groups to understand the effect on cases whose diagnosis had changed to fHP using the HP guideline. We found significant differences in the lymphocyte fraction and the presence of bird antigens between them. Moreover, there was a statistically significant difference in the CD4/CD8 ratio in the BALF of the HP + /HP + and the indeterminate groups. Although no difference between the HP + /HP + and HP-/HP + groups was found, the results of the HP-/HP + group were similar to those of the indeterminate group. Although these are interesting data, as stated in the 2020 HP guideline, the clinical factors including BALF, serum data, and antigen exposure are useful for HP diagnosis. However, none of them are fully established as a diagnostic criteria by themselves. Further research is needed to address this issue.

Eight of the cases in the HP-/HP + group were determined to be typical fHP by the guideline because of the identification of ACF and granulomas. The granulomas in these cases were not specific for HP in the original diagnosis because they were found only in the pleura or in clusters of multinucleated giant cells without epithelioid cells. The HP guidelines describe the localization of granulomas as being more common in HP, such as in the peribronchial stroma and involved with PBM. However, the cited article indicated granulomas can also be observed in the pleura and airspace ^16^. Therefore, in the present study, the presence of multiple granulomas was considered positive, even if only in the air space. If granulomas are observed only in the pleura, they should not be regarded as HP granulomas.

Of the HP-/HP + group, 46 of the 54 patients had their diagnosis changed to probable fHP due to ACF. This result suggests that the ACF determination is essential in the diagnosis of HP. One setback may be that the HP guideline does not clearly state what level of ACF should be considered significant. Eight other cases were reclassified to typical fHP, and seven of them were originally diagnosed as definite (n = 3) or probable (n = 4) UIP (Figure, Supplemental Figs. 2,3). A review of their histopathology revealed granuloma or a cluster of giant cells, but they were present in the pleura and the airspace with concurrent smoking-related changes. The diagnosis of definite UIP was made because of the dense fibrosis with architectural destruction and subpleural/ paraseptal distribution and the clear presence of fibroblastic focus. Proper use of both the IPF and HP guidelines in such cases is challenging since those pathological findings are also features of fHP. It is not uncommon for a case judged probable fHP by the HP guidelines to be judged probable UIP by the IPF guidelines, and the diagnosis of one guideline cannot be used as a basis for rejecting the other. It will be very important to reach a consensus between the HP and IPF guidelines in the future. In addition, strong smoking-related pathological findings such as emphysema were observed in 25 of the 54 patients in the HP-/HP + group. It has been reported that smoking-related fibrotic lesions are found around the airways, and the original diagnosis was not considered a finding due to fHP, but rather idiopathic IP ^20–22^. As the HP guidelines state that ACF is not a specific finding for fHP, a pathologist should decide whether to consider these 25 cases as definite or probable UIP or probable fHP. This shows that the ACF misinterpretation should be kept in mind in cases of smoking-related pathological findings.

Only three cases were indeterminate for fHP according to the 2020 HP guideline,even though the original diagnosis was HP. As shown in Fig. 3A–D, two VATS biopsies, with three biopsy sites in both cases, showed end-stage honeycomb lung with granulomas, and it was impossible to determine the presence of ACF. For TBLC, samples were obtained from two sites; however, it was inadequate to identify ACF, probably owing to its limited size (Fig. 3E–F). Regarding the three HP + /indeterminate HP cases, as ACF was not observed, the patients were considered indeterminate for fHP according to the 2020 HP guidelines. However, all of these cases had multiple poorly formed granulomas in the stroma, and the original diagnosis was HP. The judgment of one criterion, ACF, is critical for the HP guideline; however, its specificity for fHP diagnosis is unclear. A previous study demonstrated that ACF had high sensitivity but low specificity for the diagnosis of HP ^23^. Tanizawa et al. compared UIP cases with and without ACF and found that, although cases with ACF were diagnosed significantly more frequently than fHP, there was no other significant difference between them, including genetic mutations ^24^. These reports show that the clinical relevance and reproducibility of ACF are unclear. These points need to be clarified in the future.

We examined whether the HP guideline could be applied to TBLC. Of the 65 TBLC specimens reviewed, 61 cases (94%) had quality sufficient for evaluation. The results were similar to those previously reported for TBLC specimen adequacy ^25,26^. We found that the proportion of cases identified as indeterminate for HP was highest in the TBLC group. This is because findings such as ACF and granuloma are rarely observed in TBLC without sampling a large section of lung tissue. Even if fHP is suspected pathologically, the judgment with the HP guideline is often indeterminate for fHP. This shows that the judgment of samples obtained using TBLC may underestimate HP detection using the current HP guideline. Other publications have similarly demonstrated that the frequency of HP in TBLC is lower than in VATS biopsy ^27–29^. Thus, based on our experience, simply applying the HP guideline in its current form to IP samples obtained with TBLC is not recommended.

There are several limitations to this study. First, the study comprised a purely pathological assessment of the effect of the guideline on the HP diagnosis, and MDD was not performed to reach a final clinical, radiological, and pathological consensus diagnosis. However, this was not within the scope of our study, which focused on the pathological domain, and it has been addressed in a separate study ^15^. Our data may show different trends after MDD input because it is a reconciliation process by radiologists and pulmonologists, and the actual effect of pathologic discrepancy may be diminished. Second, the study was retrospective in design, meaning we were restricted to the available data. Third, we used cases from a single center, which may have introduced some selection bias and composed of one MDD group. Fourth, the location of granuloma in the lung defined by HP guideline may be interpreted differently by different raters. In the future, it is necessary to examine whether the diagnosis of fHP differs depending on the location of granuloma. Nevertheless, this is the first study to address the impact of the newly introduced HP guideline in a large case series.

We confirmed that the pathological criteria of the 2020 HP guideline efficiently excluded most non-HP cases^11^. Concurrently, approximately one-fourth of all cases of fibrotic IP diagnostically changed from not HP to fHP. The significance of our results should be further evaluated and validated to clarify if such changes convey clinical and prognostic significance are an improvement or deterioration for the patients.

## Supplementary Information

Supplementary Information.

## Data Availability

Data and materials of this work are available from the corresponding author upon reasonable request.
