# Supplementary material for: Effect of the 2020 hypersensitivity pneumonitis guideline on the pathologic diagnosis of interstitial pneumonia
Source: Sci Rep. 2023 Jun 8;13:9318. doi: 10.1038/s41598-023-35986-9 (PMC10250339; doi:10.1038/s41598-023-35986-9)
Supplement: Supplementary file 1 — Supplementary Information. [file 41598_2023_35986_MOESM1_ESM.pptx]

## Slide 1
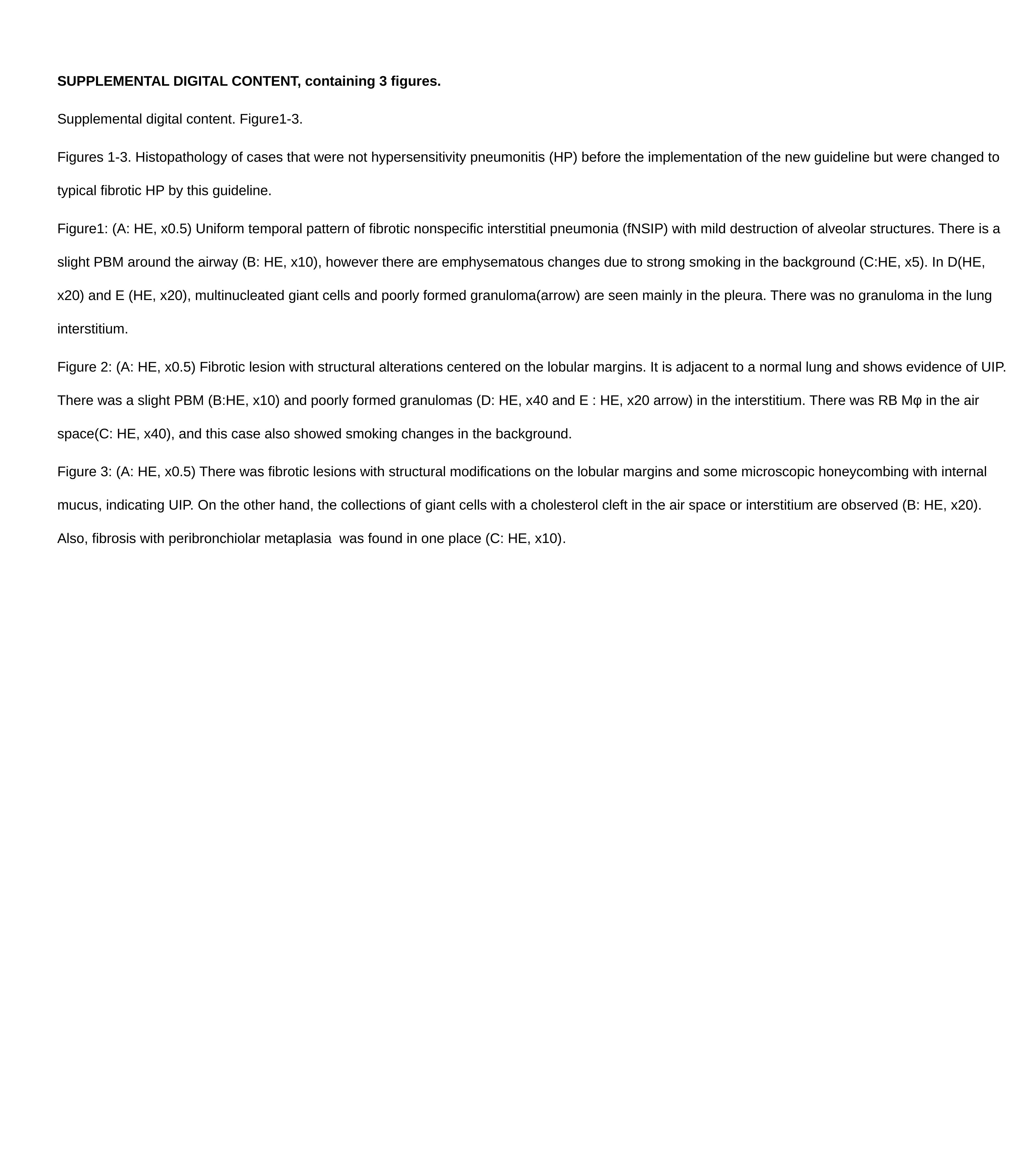

SUPPLEMENTAL DIGITAL CONTENT, containing 3 figures.
Supplemental digital content. Figure1-3.
Figures 1-3. Histopathology of cases that were not hypersensitivity pneumonitis (HP) before the implementation of the new guideline but were changed to typical fibrotic HP by this guideline.
Figure1: (A: HE, x0.5) Uniform temporal pattern of fibrotic nonspecific interstitial pneumonia (fNSIP) with mild destruction of alveolar structures. There is a slight PBM around the airway (B: HE, x10), however there are emphysematous changes due to strong smoking in the background (C:HE, x5). In D(HE, x20) and E (HE, x20), multinucleated giant cells and poorly formed granuloma(arrow) are seen mainly in the pleura. There was no granuloma in the lung interstitium.
Figure 2: (A: HE, x0.5) Fibrotic lesion with structural alterations centered on the lobular margins. It is adjacent to a normal lung and shows evidence of UIP. There was a slight PBM (B:HE, x10) and poorly formed granulomas (D: HE, x40 and E : HE, x20 arrow) in the interstitium. There was RB Mφ in the air space(C: HE, x40), and this case also showed smoking changes in the background.
Figure 3: (A: HE, x0.5) There was fibrotic lesions with structural modifications on the lobular margins and some microscopic honeycombing with internal mucus, indicating UIP. On the other hand, the collections of giant cells with a cholesterol cleft in the air space or interstitium are observed (B: HE, x20). Also, fibrosis with peribronchiolar metaplasia was found in one place (C: HE, x10).

## Slide 2
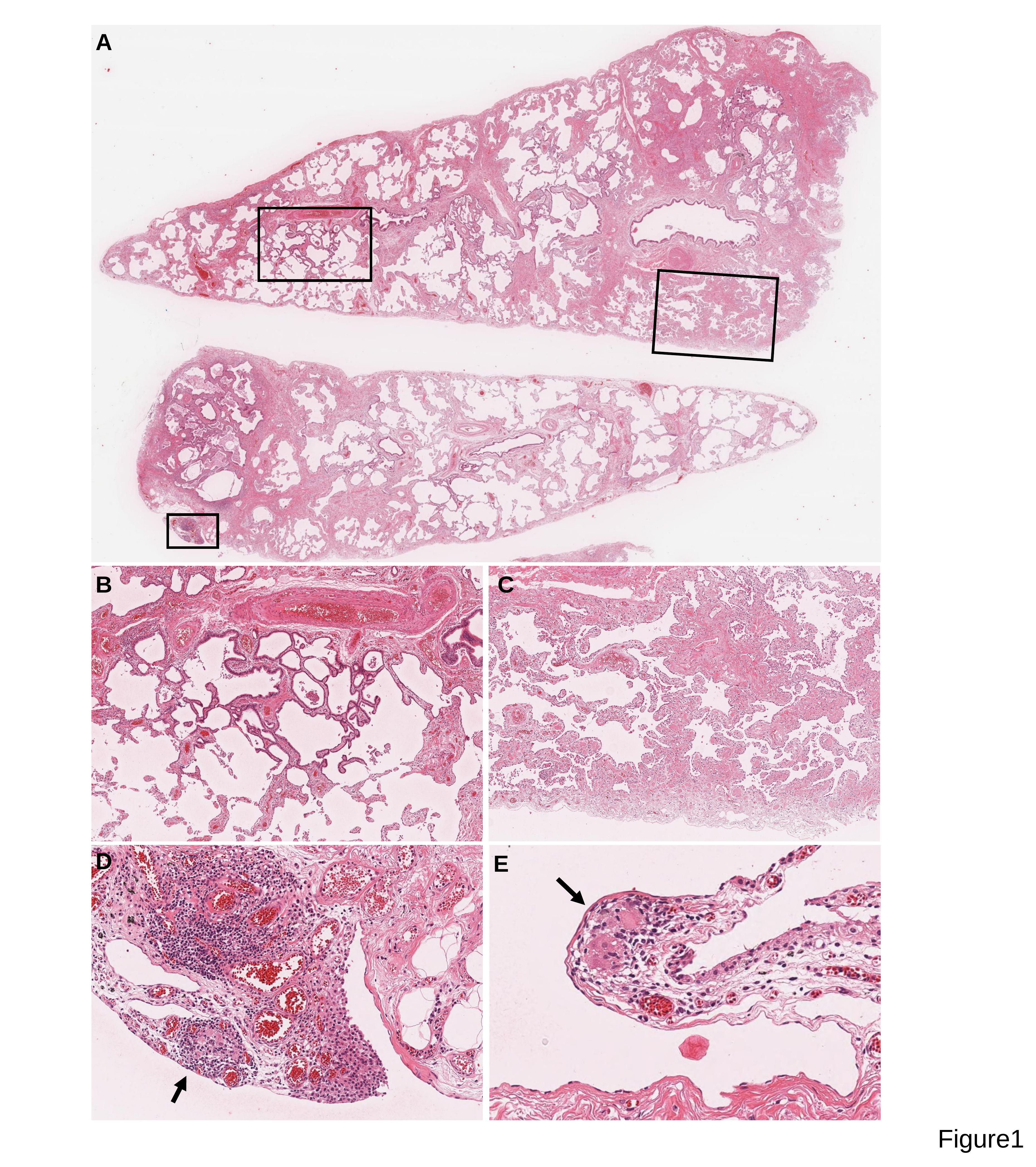

A
B
C
D
E
Figure1

## Slide 3
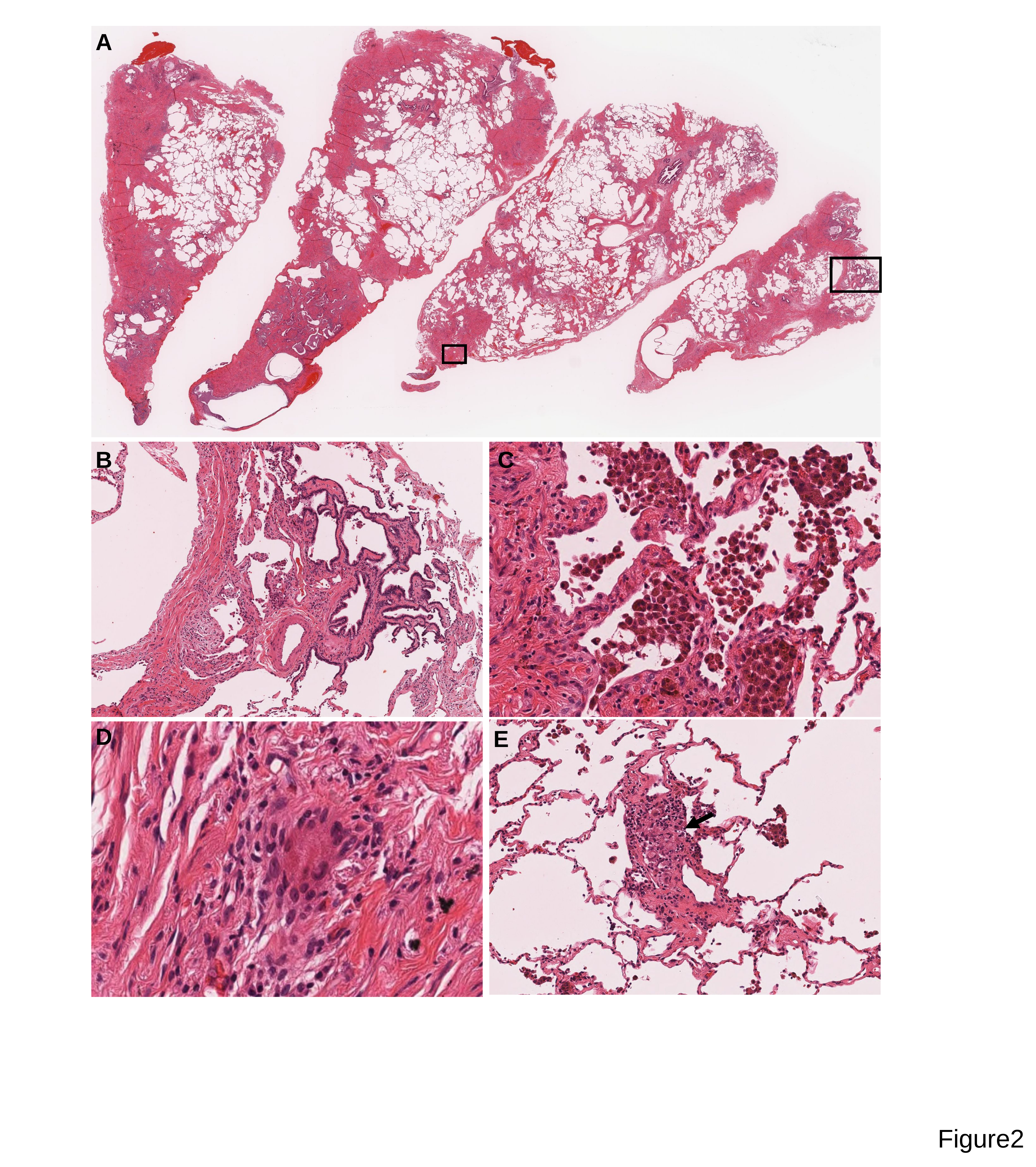

A
B
C
D
E
Figure2

## Slide 4
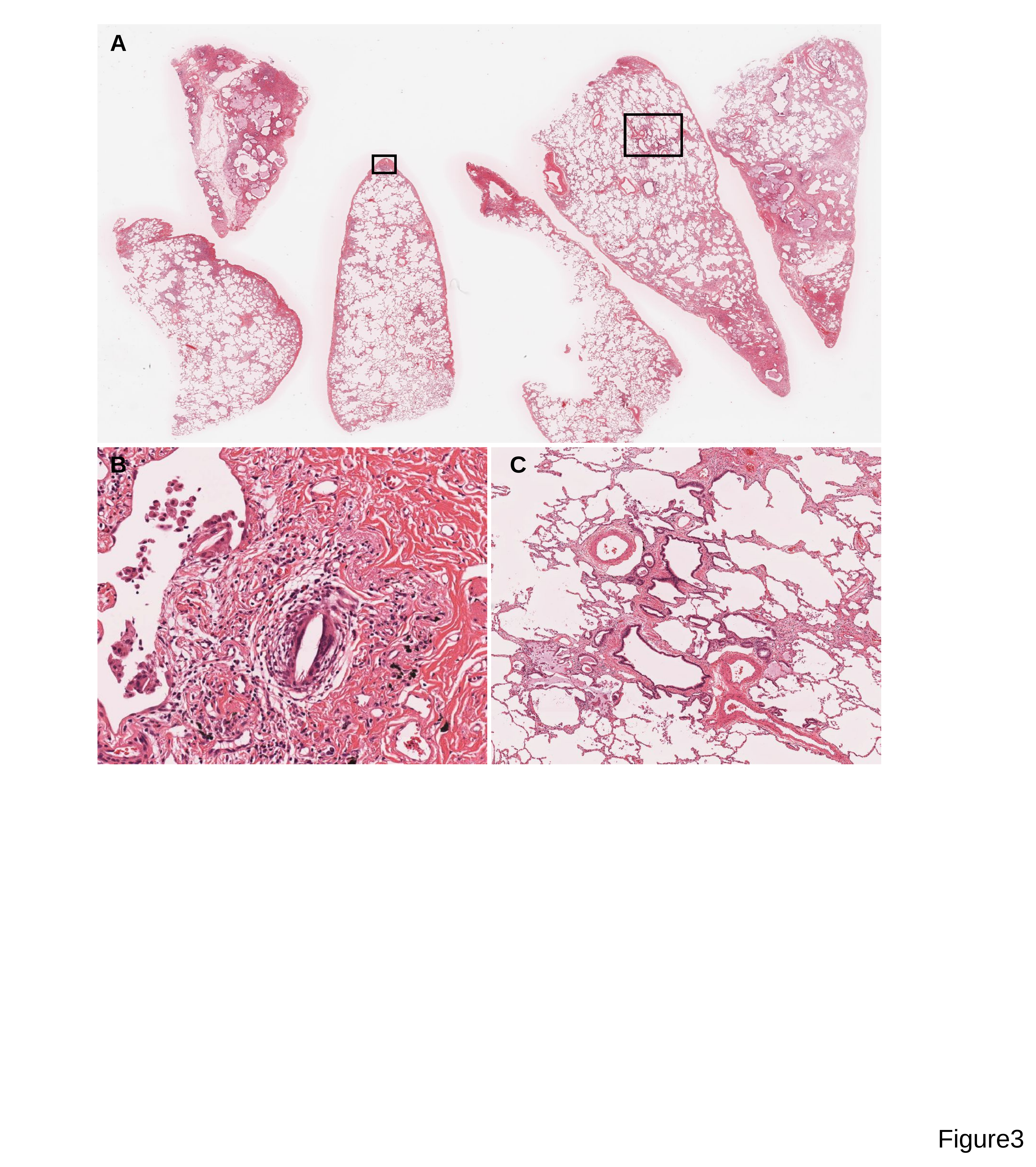

A
B
C
Figure3
